# Antibacterial intraosseous implant surface coating that responds to changes in the bacterial microenvironment

**DOI:** 10.3389/fbioe.2022.1016001

**Published:** 2023-01-09

**Authors:** Xin Bai, Jiawei Yu, Jie Xiao, Yanping Wang, Zhe Li, Hao Wang

**Affiliations:** ^1^ Jiande First People’s Hospital, Hangzhou, Zhejiang, China; ^2^ Zhuji Affiliated Hospital of Wenzhou Medical University, Shaoxing, Zhejiang, China; ^3^ Department of Orthopedics, Quanzhou First Hospital Affiliated of Fujian Medical University, Quanzhou, Fujian, China

**Keywords:** infection, antibacterial coating, *Staphylococcus aureus*, layer-by-layer, microenvironment

## Abstract

Bone implant-associated infection is one of the most challenging problems encountered by orthopedic surgeons. There is considerable interest in the development of drug-loaded antibacterial coatings for the surfaces of metal implants. However, it is difficult to achieve the stable local release of an effective drug dose for many antibacterial coatings. In the present study, analyses of the thickness and water contact angle of multiple layers confirmed the successful assembly of multilamellar membrane structures. Measurement of the zone of bacterial inhibition indicated gradual degradation of the (montmorillonite [MMT]/hyaluronic acid [HA])_10_ multilamellar film structure with concentration-dependent degradation during incubation with hyaluronidase solution and *Staphylococcus aureus*. *In vivo* results resembled the *in vitro* results. Overall, the findings confirm that the (MMT/HA-rifampicin)_10_ multilamellar film structure exhibits good antibacterial properties and excellent biocompatibility. Further studies of the clinical potential of the antibacterial coating prepared in this experiment are warranted.

## 1 Introduction

Since the introduction of various metallic and non-metallic fixation materials in the field of orthopedics, and particularly with the development of internal and external fixation over the past century, advances in materials science and medical techniques have led to the increasing use of orthopedic surgery (Berger et al., 2016; Li et al., 2017). However, bone and soft tissue infections remain among the most challenging complications of orthopedic surgery (Neumann et al., 2016; Pickett et al., 2018). In cases of open fractures, various fixation devices (e.g., plate screws, external fixation brackets, and intramedullary nails) may contribute to infection (Amerstorfer et al., 2017; Perez-Jorge et al., 2017). Closed fractures have infection rates of approximately 1.5%, whereas open fractures have infection rates of 3–40% (Neumann et al., 2016; Kendall and Gorgone, 2018). The incidence of trauma-related open fractures is increasing (Tessier et al., 2016). Approximately 60% of open fractures are contaminated by bacteria at the time of injury (Caroom et al., 2018). Fixation-related infections can also cause serious adverse consequences for patients, potentially requiring systemic antibiotics, multiple debridement-and-revision surgeries, extended hospital stays, and/or amputations; they may also result in death (Barnett et al., 2016; Klein et al., 2016). Therefore, fixation-related infections have become a major research focus in the fields of orthopedics, microbiology, biomaterials, and drug development (Jones et al., 2016; Sugii et al., 2017).

The local preventive or therapeutic application of antibiotics has received considerable attention for the treatment of internal fixation-related infections (Flamant et al., 2016). Intravenous infusion of antibiotics based on the results of drug sensitivity testing is a common clinical treatment method, but systemic drug administration can only achieve low concentrations in lesions while potentially damaging areas such as the liver, kidney, and ears (Girmenia and Iori, 2017; Kyriakidis et al., 2017). Therefore, the application of drug-loaded antibacterial coatings on the surfaces of metal implants has been proposed to aid in preventing infection and avoiding side effects associated with systemic administration (Li et al., 2016). Jennings et al. (2015) mixed vancomycin and amikacin into lecithin to form a uniform solution that could coat the surface of titanium metal. They found that the drugs could be stably released for approximately six days, and their system exhibited *in vitro* antibacterial activity against *Staphylococcus aureus*. Braem et al. (2015) used titanium with a pore structure as a drug carrier, then integrated it into a mesoporous SiO_2_ diffusion barrier to control drug release on the titanium metal surface. Although this method resolved the problem of drug burst release, the drug release time remained short. Hirschfeld et al. (2017) used plasma vapor deposition to deposit vertically aligned carbon nanotubes approximately 700 nm long and 10–200 nm in diameter on the surface of titanium; the tubes were used to carry rifampicin (RFP). Their experiments confirmed the antibacterial ability of the RFP from these carbon nanotubes against *Staphylococcus epidermidis.* The greatest limitation of using these surface coatings as antibacterial drug carriers is that they generally do not allow the stable local release of effective drug doses. There is often a burst release in the early stages of drug release, and it is difficult to maintain accurate drug release for an extended duration. There has been considerable progress in the construction of stimulus-sensitive drug delivery systems on the surfaces of biological materials (Huang et al., 2016; Wang et al., 2016), and numerous drug delivery systems based on enzymes, temperature, and pH sensitivity have been constructed (Sun et al., 2021). These stimulus-sensitive drug delivery systems can control drug release and avoid drug resistance caused by excessive short-term release or minimal long-term release. The microenvironment of bacterial infection has multiple unique biological characteristics, including the abnormal expression of some enzymes and the specific release of toxins (Wang et al., 2018a). These specific biological characteristics can be used to prepare smart drug delivery systems with appropriate microenvironmental responses to ensure controlled release and accumulation of antibacterial drugs at the site of infection (Aycan and Alemdar, 2018). Layer-by-layer self-assembly based on multisubstance interactions is a useful method for the formation of nanostructured multilamellar films (Chen et al., 2017). External stimuli can achieve the selective embedding of substances and the intelligent controlled release of drugs from multilamellar films (Mable et al., 2018).

*S. aureus* is the main species of pathogenic bacteria encountered after surgery or trauma and the primary species involved in endosseous implant-related bone, joint, and soft tissue infections (Gundtoft et al., 2017; Araújo et al., 2018). The entry of *S. aureus* into lymphatic vessels or blood can cause sepsis. RFP, which has high antibacterial activity against various Gram-positive bacteria (Branch-Elliman et al., 2017), is a key antibiotic for treating severe *S. aureus* infections. It has an important role in clinical therapy and is an ideal antibiotic for topical application (Downes et al., 2017). During bacterial infection, the surrounding environment secretes large amounts of hyaluronidase (HAS) and phospholipase (Seroussi et al., 2018; Liu et al., 2021). Montmorillonite (MMT)/hyaluronic acid (HA) multilamellar film actively disintegrates in the presence of infection (Wang et al., 2018b). MMT is a typical clay mineral with a unique crystal structure that provides it with a high cation exchange capacity and a high expansion capacity; thus, it is widely used in various fields (Enteshari Najafabadi and Bagheri, 2017). Because of its high specific surface area, MMT can additionally be included in multilamellar films to load drugs at high concentrations and prevent rapid release (Qiao et al., 2016; Chabbi et al., 2018). HA is an acidic mucopolysaccharide that is widely distributed in the human body. It solubilizes readily in water, where it forms an acidic solution with a considerable negative charge. HA has good biocompatibility and various clinical uses (e.g., as a lubricant in joint surgery and a moisturizing agent in eye surgery) (Friedrich and Washburn, 2017; Hwang et al., 2017). In the present study, drug-loaded polyelectrolyte multilayers of (MMT/HA-RFP)_10_ were fabricated in a layer-by-layer manner and exhibited HAS release-induced degradation in the presence of infection.

## 2 Materials and methods

### 2.1 Reagents and materials

MMT was obtained from Zhejiang Sanding Technology Co., Ltd. (Zhejiang, China). HA was purchased from Freda Biochem Co., Ltd. (Shandong, China). RFP and polyethyleneimine (Mw: 25 kDa) were purchased from Sigma-Aldrich (St. Louis, MO, United States). Titanium Kirschner wires (K-wires; 1.25 mm) were purchased from MK Medical GmbH & Co. (Emmingen-Liptingen, Baden-Württemberg, Germany). Luria-Bertani agar and Luria-Bertani broth were purchased from Difco Laboratories (Detroit, MI, United States).

### 2.2 Construction of polyelectrolyte multilayers

The substrates were ultrasonically cleaned in acetone and in ethanol for 2 h each, then dried with compressed air. MMT stock solution (0.5 mg/ml, pH = 2.5) was prepared. Next, HA and RFP were separately dissolved at a concentration of 1.0 mg/ml in deionized water. Before assembly, the reaction solutions were dispersed by ultrasonication for 2 h. All substrates were subsequently deposited in polyethyleneimine solution (3 mg/ml, 20 min) as a precursor, then immersed alternately in MMT solution and HA-RFP solution for 15 min each at room temperature. Finally, the obtained hybrid multilamellar films were dried under a stream of nitrogen gas. This immersion cycle corresponded to the deposition of one bilayer. The above steps were repeated 10 times to yield (MMT/HA-RFP)_10_ multilamellar membrane structures.

### 2.3 Characterization of materials

We tested the following polymer coatings: (MMT/HA-RFP)_0.5_, (MMT/HA-RFP)_3_, (MMT/HA-RFP)_7_, (MMT/HA-RFP)_10_, and (MMT/HA)_10_. The surface roughness of silicon wafers, the substrate material, was measured by atomic force microscopy. Additionally, scanning electron microscopy (SEM) was performed to examine the surface morphology of each sample. To determine the wetting properties of the multilamellar film surfaces, water contact angles were measured using a video optical contact angle measuring device (OCA15pro; DataPhysics Co., Filderstadt, Germany) in air at room temperature (25°C). The mean value of each multilamellar film structure was calculated from the measurements at three different locations. To confirm the successful assembly of multilamellar membrane structures, the thickness of (MMT/HA-RFP)_n_ deposited in each layer was measured by spectroscopic ellipsometry (M-2000 DITM; J.A. Woollam, Lincoln, NE, United States).

### 2.4 Zone of inhibition measurement and SEM

Glass slides were selected as the substrate material in this study. Layer-by-layer self-assembled multilamellar films were prepared using the method described above. The multilamellar films were immersed for two days in 0.01 M phosphate-buffered saline (PBS), HAS solution, and *S. aureus* solution. At the end of the soaking period, all samples were removed and dried in an oven. *S. aureus* was grown from broth on nutrient agar, then plated on Luria-Bertani agar. Growth inhibition was verified based on the zone of inhibition (ZOI) after incubation at 37°C for 24 h. SEM was used to examine changes in the (MMT/HA-RFP)_10_ multilamellar coating on the surface of the glass slides.

### 2.5 Assessment of biofilm formation

Unmodified silicon wafers, as well as (MMT/HA)_10_ and (MMT/HA-RFP)_10_ multilamellar film silicon wafers, were prepared as three parallel samples, then immersed in 2 ml of *S. aureus* suspension [10 colony-forming units/ml (Kendall and Gorgone, 2018)] and incubated at 37°C for 5, 10, or 15 h. At preset time points, the surfaces were thoroughly rinsed with PBS. Next, all samples were stained with 1% (wt/vol) crystal violet solution for 15 min, then washed three times with PBS. Finally, 1 ml of 95% (v/v) ethanol was added to each well. The mixture was then incubated for 15 min in the dark and shocked for 10 s every 5 min at room temperature. The absorbance at 570 nm (A570) was measured with a microplate reader (BioTek, Winooski, VT, United States).

### 2.6 Shaking flask culture and live/dead staining assay

The shaking flask culture method was used to comprehensively evaluate the *in vitro* antibacterial properties of the coating. (MMT/HA-RFP)_10_-coated polydimethylsiloxane was immersed in *S. aureus* solution and cultured. Aliquots of the bacterial solution were collected at specified time points (2 h, 4 h, 6 h, 8 h, 10 h, and 12 h) and diluted; next, they were evenly spread on agar plates, cultured, and counted. For direct analysis of the live/dead populations of bacteria on the surface of (MMT/HA-RFP)_10_ multilamellar membrane structures, fluorescence microscopy was conducted with live/dead staining. (MMT/HA-RFP)_10_-coated glass slides and unmodified glass slides were co-cultured with 3 ml of *S. aureus* solution, incubated at 37°C for 6, 12, or 24 h, and then subjected to live/dead staining.

### 2.7 *In vitro* hemolysis rate

Four ml of fresh anticoagulated rat blood was diluted with 5 ml of normal saline. The multilamellar film was incubated in 10 ml of normal saline (37°C constant temperature water bath, 30 min). Next, 0.2 ml of diluted blood was added by mixing, and the solution was incubated at a constant temperature of 37°C for 60 min. Aliquots of 500 μL were collected and centrifuged at 1,500 rpm for 5 min. The absorbance of the resulting supernatant at 540 nm was measured with an ultraviolet-visible spectrophotometer (PuXi TU-1800; China). In the positive control group, 0.2 ml of diluted blood was added to 10 ml of distilled water; in the negative control group, 0.2 ml of diluted blood was added to 10 ml of 0.9% NaCl solution.

Hemolysis of the multilamellar film was calculated using the following equation:

Hemolysis rate % = (ODs − ODn)/(ODp − ODn) × 100

where ODs, ODp, and ODn represent the respective absorbance values of the sample, negative control, and positive control groups.

### 2.8 Animal experiments

#### 2.8.1 Biocompatibility analysis

Thirty male rats (body weight 200 ± 20 g) were obtained from SLAC Laboratory Animal Co., Ltd. (Shanghai, China) for use in this experiment. All experiments were approved by the Animal Research Ethics Committee of the Second Affiliated Hospital (Jiande Branch) of the School of Medicine, Zhejiang University. All rats were housed in an animal room with controlled humidity and temperature, where they were provided with adequate food and water. Unmodified, (MMT/HA)_10_-coated, and (MMT/HA-RFP)_10_ multilamellar film-coated K-wires were prepared. The rats were randomly divided into three groups (*n* = 10 per group). For the experimental analysis, the rats were initially anesthetized with 10% chloral hydrate (0.35 ml/100 g). The left knee of each rat was shaved, the overlying fur was removed, and the skin was disinfected. Then, the skin and muscles were cut to expose the tibial plateau. During this procedure, neurovascular injury was avoided when possible. Under sterile conditions, a 0.8-mm K-bit was used to drill vertically into the medullary cavity, and a sterile syringe was used to aspirate bone marrow from the tibia. Unmodified, (MMT/HA)_10_-coated, or (MMT/HA-RFP)_10_ multilamellar film-coated K-wires were placed in the boreholes according to each rat’s group designation. The drilled hole was sealed with bone wax, and the incision was sutured layer by layer. After each rat had regained consciousness, it was maintained in an appropriate environment with adequate food and water. All rats were sacrificed 4 weeks after surgery. Their tissues (lung, kidney, heart, and liver) were cryopreserved, cut into sections, fixed, and stained with hematoxylin and eosin (H&E), in accordance with standard protocols.

#### 2.8.2 *In vivo* antibacterial experiments

Thirty additional rats were used to determine the *in vivo* antimicrobial efficacy of the multilamellar film structure. The animals were divided randomly into three groups (*n* = 10 per group): 1) an unmodified group, with placement of unmodified K-wires and injection of 10 µL of 10^6^ *S. aureus* cells/mL; 2) a (MMT/HA-RFP)_10_ group, with placement of (MMT/HA-RFP)_10_-coated K-wires and injection of 10 µL of 10^6^ *S. aureus* cells/mL; and 3) a sham group, with placement of unmodified K-wires and without injection of *S. aureus*. For each animal, the drilled hole was sealed with bone wax, and the incision was sutured layer by layer. After each rat had regained consciousness, it was maintained in a standard environment with adequate food and water. The condition of each wound was monitored regularly.

##### 2.8.2.1 Measurement of body temperature and inflammatory index *in vivo*

At regular intervals, each rat’s body temperature was monitored and their blood collected from the tail tip to examine white blood cell (WBC) count, as well as levels of high-sensitivity C-reactive protein (hs-CRP), interleukin (IL)-1β, IL-6, and IL-8.

##### 2.8.2.2 Colonies from K-wires and soft tissue

Left tibia specimens were thawed at room temperature, then snap-frozen in liquid nitrogen. The soft tissue was pulverized in sterile PBS for 60 s. Concurrently, the K-wires were sonicated in sterile PBS for 30 min. All procedures were performed in a sterile environment. The fluids from soft tissue and K-wires were diluted in sterile saline, inoculated onto agar plates, and placed in a bacteriological incubator at a constant temperature of 37°C. Finally, the numbers of bacterial colonies on agar plates were counted.

##### 2.8.2.3 X-ray examination

Each rat was euthanized and their left tibia extracted for X-ray examination (current, 250 mA; energy, 45 kV; integration time, 200 ms) (Carestream DRX; Carestream, Rochester, NY, United States). Three deputy chief orthopedic physicians evaluated the tibia destruction, osteolytic lesions, and tissue surrounding the tibia. Then, each tibia was wrapped in saline-saturated gauze and stored at −20°C.

##### 2.8.2.4 Computed tomography examination

Left tibia specimens were thawed at room temperature and their soft tissue removed. Next, the samples were fixed with 4% paraformaldehyde in PBS for 12 h. Tibia lesions were observed by CT (energy, 70 kV at 114 μA; integration time, 300 ms; threshold, 220) (Skyscan 1,173; Skyscan, Kontich, Belgium). A ring with a surface radius of 0.1 mm from the metal implant was evaluated as the volume of interest. CT software was used to analyze bone mineral density (BMD), trabecular bone number (Tb.N), percentage bone volume (BV/TV), and trabecular thickness (Tb.Th); three-dimensional histograms were also constructed.

##### 2.8.2.5 Tibia specimen bending test

Left tibia specimens were thawed at room temperature and their soft tissue removed. The specimens were subjected to three-point bending tests to determine their integration strength, using an ElectroForce 3200 computer-controlled testing machine (Bose Corp., Eden Prairie, MN, United States); a crosshead speed of 1 mm min−1 was applied until each specimen broke. Finally, the maximum load, maximum strain, maximum bending moment, maximum stress, and bending section modulus were determined for the quantitative analysis of the mechanical strength of each tibia.

##### 2.8.2.6 Histological analysis

Lesions of the tibial metaphysis were fixed with 4% paraformaldehyde for 48 h. Tibia bone specimens were decalcified with ethylenediaminetetraacetic acid solution for one month, then embedded in paraffin. The specimens were cut into 5-μm-thick sections; stained with H&E, Masson’s trichrome, and toluidine blue; and then observed under a microscope.

### 2.9 Statistical analysis

Data analyses were performed using SPSS 24.0 (SPSS Inc., Chicago, IL, United States). Multisample means were evaluated by one-way analysis of variance (α = 0.05). Comparisons among groups were analyzed by the *t*-test. In all analyses, *p* < 0.05 was considered to indicate statistical significance.

## 3 Results and discussion

### 3.1 Characterization of materials

**FIGURE 1:**
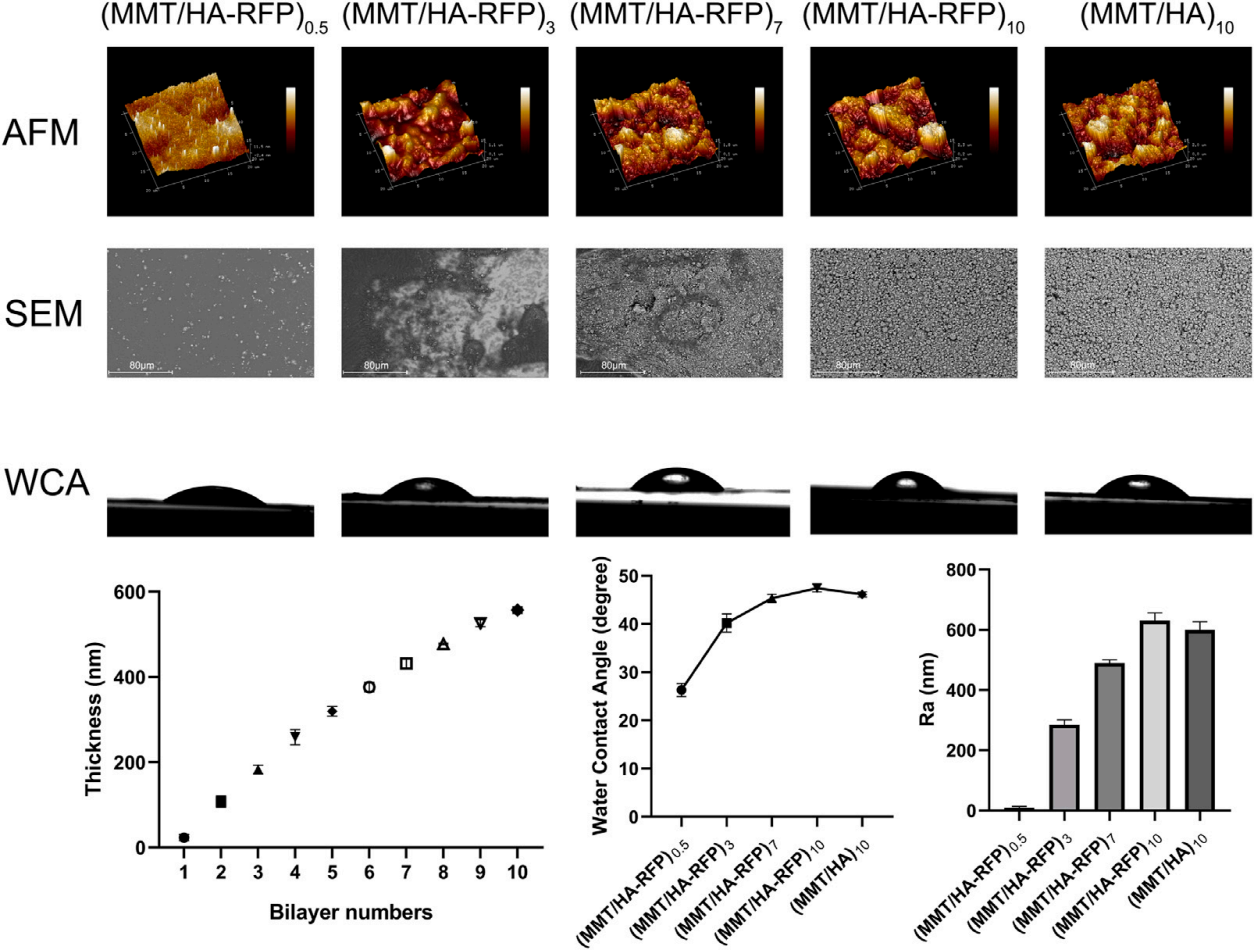
Atomic force microscopy (AFM), SEM, water contact angle (WCA), and thickness analysis of multilamellar membrane structures during co-assembly.

As shown in Figure 1, we measured the roughness of surfaces using atomic force microscopy (AFM). After the coating was deposited, surface roughness increased. More specifically, the (MMT/HA-RFP)_0.5_ coating had a roughness of 8.70 ± 4.14 nm, and the (MMT/HA-RFP)_3_ coating had a roughness of 285.33 ± 12.71 nm. The roughness of the (MMT/HA-RFP)_7_ coating was 489.33 ± 9.03 nm, that of the (MMT/HA-RFP)_10_ coating was 630.00 ± 21.74 nm, and that of the (MMT/HA)_10_ coating was 599.67 ± 22.07 nm. Using SEM, we observed multiple layers on the surface of substrate, with uniform membrane structure. Next, the contact angle was measured to determine the hydrophilicity of each surface. The contact angles were 26.30 ± 1.10° for the (MMT/HA-RFP)_0.5_ coating, 40.20 ± 1.56° for the (MMT/HA-RFP)_3_ coating, 45.37 ± 0.63° for the (MMT/HA-RFP)_7_ coating, and 47.43 ± 0.63° for the (MMT/HA-RFP)_10_ coating. In addition, the contact angle of the (MMT/HA)_10_ coating was 46.13 ± 0.42°. This shows that the material has a hydrophilic surface after coating modification. Many reports have indicated that moderate hydrophilicity could increase the biocompatibility of the material (Bluestein et al., 2017; Hu et al., 2017). Finally, we performed thickness measurements on multilayer structures. The thickness of the multilamellar membrane structures clearly exhibited linear growth as assembly progressed. More specifically, the thickness of the (MMT/HA-RFP)_1_ coating was 23.33 ± 5.79 nm. As self-assembly progressed, the (MMT/HA-RFP)_5_ coating had a thickness of 319.67 ± 9.46 nm and the (MMT/HA-RFP)_10_ coating had a thickness of 557.00 ± 5.10 nm.

### 3.2 ZOI measurement and SEM

**FIGURE 2:**
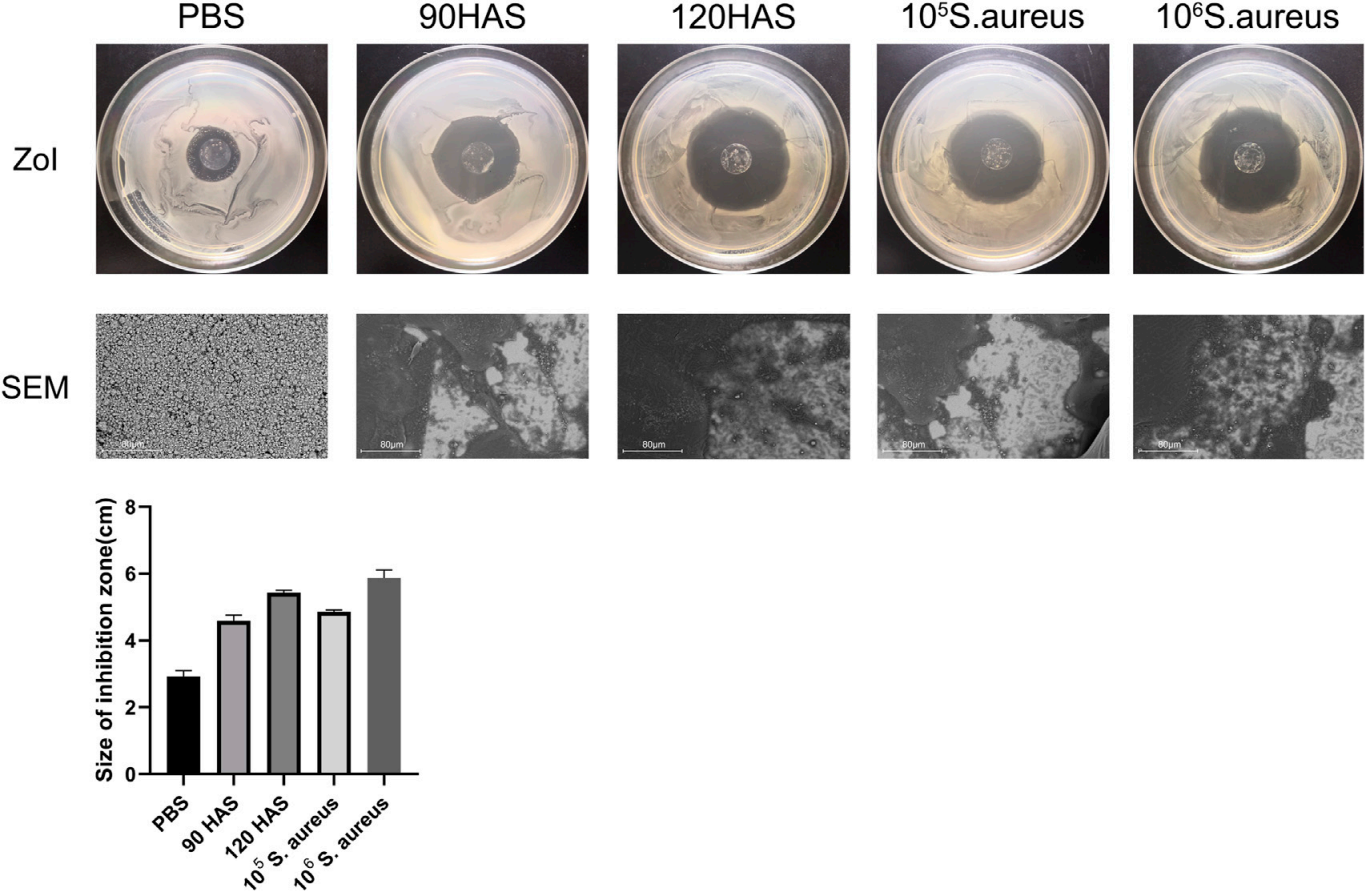
ZOI and SEM of (MMT/HA-RFP)_10_ multilamellar film-coated glass slides after immersion in 0.01 M PBS, HAS, and *Staphylococcus aureus* solutions.

The ZOI represents the diameter of the bacteriostatic area, which reflects the antibacterial coating of the multilamellar films. As shown in Figure 2, after immersion in 0.01 M PBS, the ZOI around the multilamellar films was 2.93 ± 0.14 cm, whereas it was 4.59 ± 0.14 cm and 5.43 ± 0.06 cm after immersion in HAS solutions at concentrations of 90 and 120 U/mL, respectively. After multilamellar films had been immersed in solutions containing 105 or 106 colony-forming units/mL of *S. aureus*, the ZOIs were 4.86 ± 0.05 cm and 5.87 ± 0.20 cm, respectively. Changes in the multilamellar coating on the surfaces of glass slides were examined by SEM. Compared with immersion in PBS, the degree of disintegration of the (MMT/HA-RFP)_10_ multilamellar film structure gradually increased after two days of immersion in solutions with increasing concentrations of HAS and *S. aureus*. The ZOI increase with increasing concentrations of HAS and bacterial solutions may have occurred because the HAS secreted by *S. aureus* promoted the disintegration of the multilamellar film, leading to large amounts of disintegrated film fragments on the surface of the glass slides, and MMT could be loaded with large amounts of the test drug.

### 3.3 Determination of biofilm formation

**FIGURE 3:**
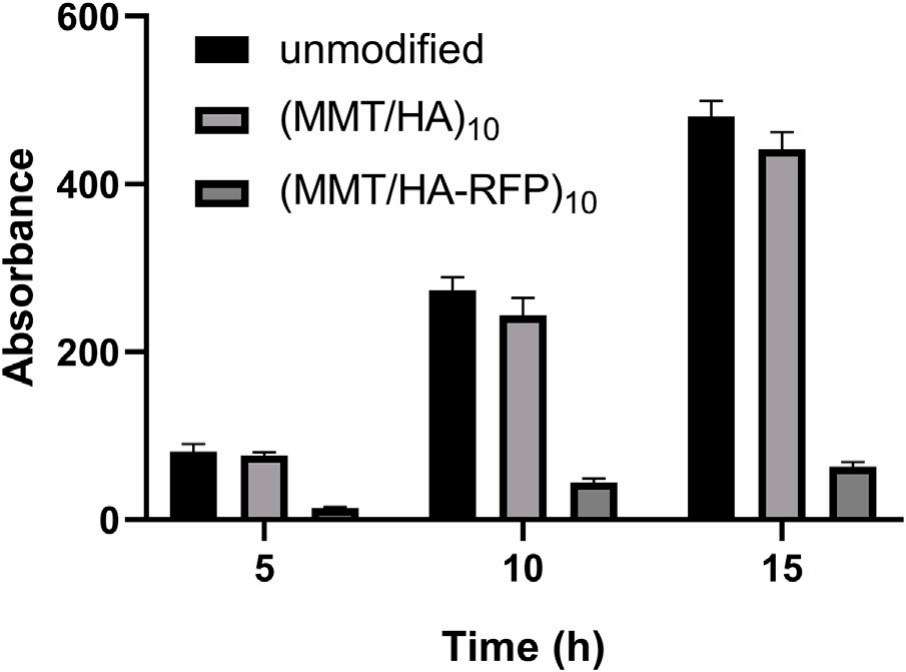
Determination of biofilm formation after immersion with unmodified, (MMT/HA)_10_-coated, and (MMT/HA-RFP)_10_ multilamellar film-coated silicon wafers at various time points.

Biofilm inhibition activity was assessed by crystal violet staining (Figure 3). The crystal violet absorption by the (MMT/HA-RFP)_10_ samples was substantially lower than the crystal violet absorption by the unmodified samples at all time points examined (5, 10, and 15 h). With increasing culture time, the crystal violet absorption by all (MMT/HA-RFP)_10_ samples slowly increased, whereas the crystal violet absorption by unmodified samples substantially increased, confirming that the (MMT/HA-RFP)_10_ multilamellar film structure had strong biofilm-inhibition effects.

### 3.4 Shaking flask culture and live/dead staining assay

**FIGURE 4:**
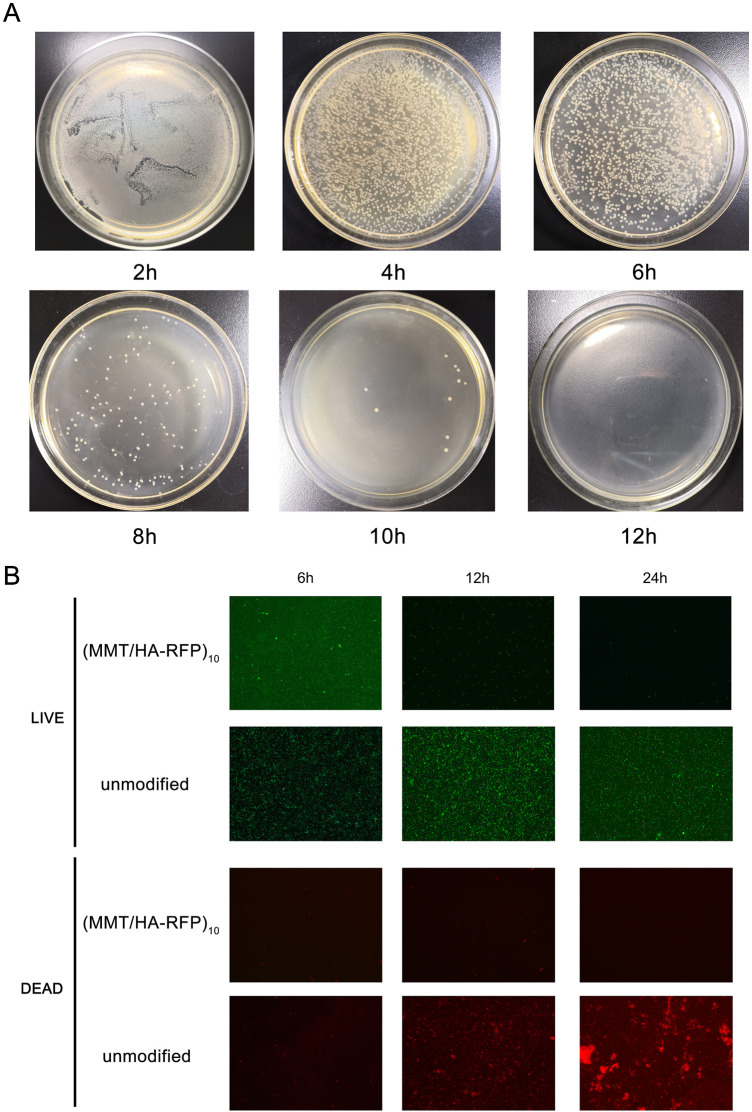
**(A)** Shaking flask culture and **(B)** live/dead staining assay.

The number of bacteria was calculated at various time points using the shaking flask method. As shown in Figure 4, the number of bacteria decreased over time, nearly reaching zero at 12 h, indicating that the antibacterial coating can rapidly and efficiently sterilize bacterial solutions. After 6, 12, and 24 h of coincubation, the numbers of both live (green) and dead (red) bacteria were substantially reduced on the surface of the (MMT/HA-RFP)_10_ multilamellar film, compared with the unmodified group. These observations indicate that the (MMT/HA-RFP)_10_ polymer multilamellar film has excellent antibacterial properties. The (MMT/HA-RFP)_10_ multilamellar membrane structure may also remove some dead bacteria from the surface.

### 3.5 *In vitro* hemolysis rate

**FIGURE 5:**
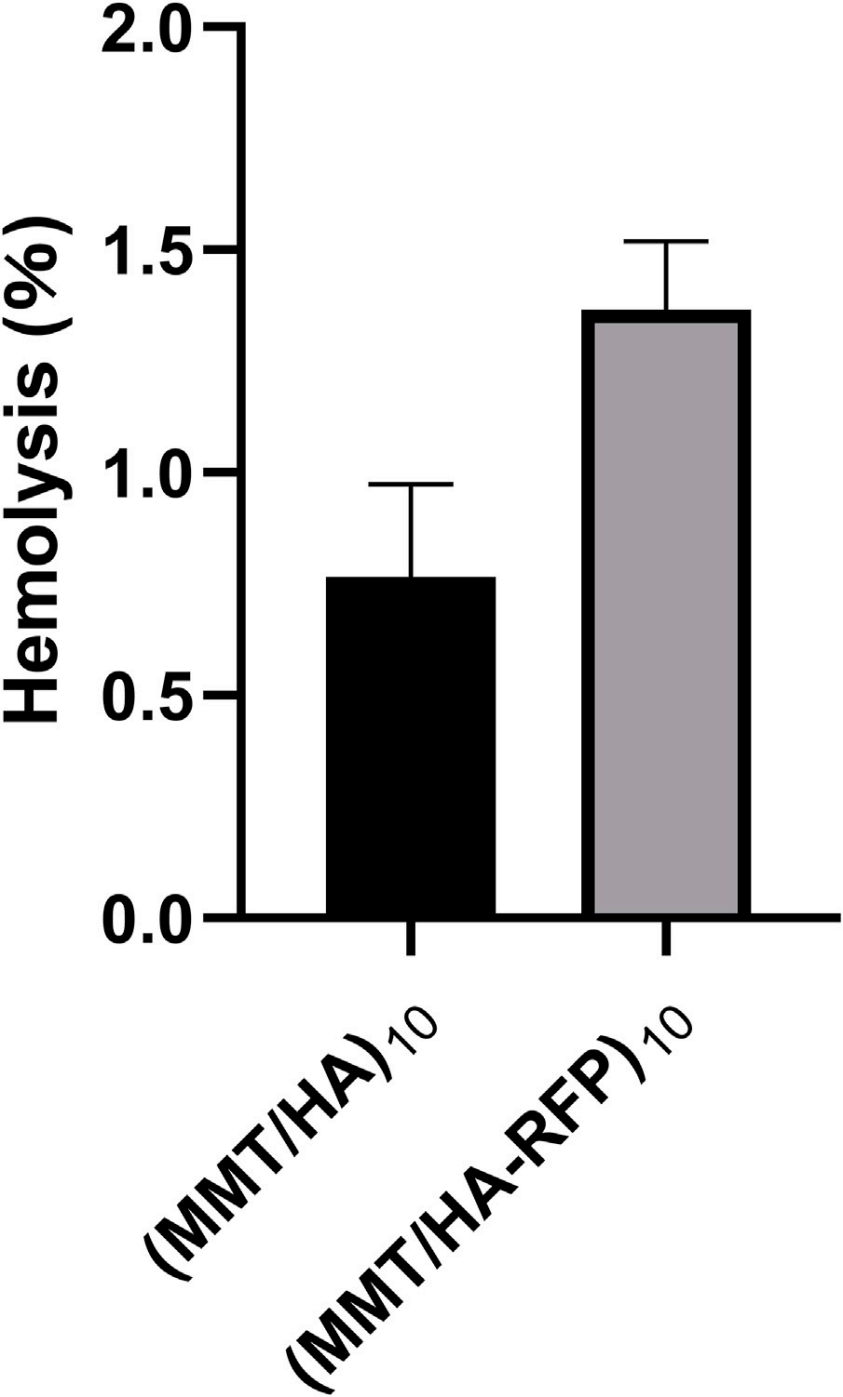
Hemolysis rates of (MMT/HA)_10_ and (MMT/HA-RFP)_10_ multilamellar films.

The hemolysis rate represents the degree of damage to red blood cells caused by the test material, which is an important consideration in the experimental analysis of medical materials (Figure 5). The absorbance value of the positive control group (ODp) was 1.225 L/g/cm, whereas the absorbance value of the negative control group (ODn) was 0.001 L/g/cm. The absorbance values of the sample (ODs) were substituted into the formula specified in the Methods to calculate the hemolysis rate. The hemolysis rate of the membrane surface was <5%, consistent with the standard for medical polymer materials, and the multilayer structure fulfilled the requirements of the body environment (Dang et al., 2019).

### 3.6 Biosafety evaluation

**FIGURE 6:**
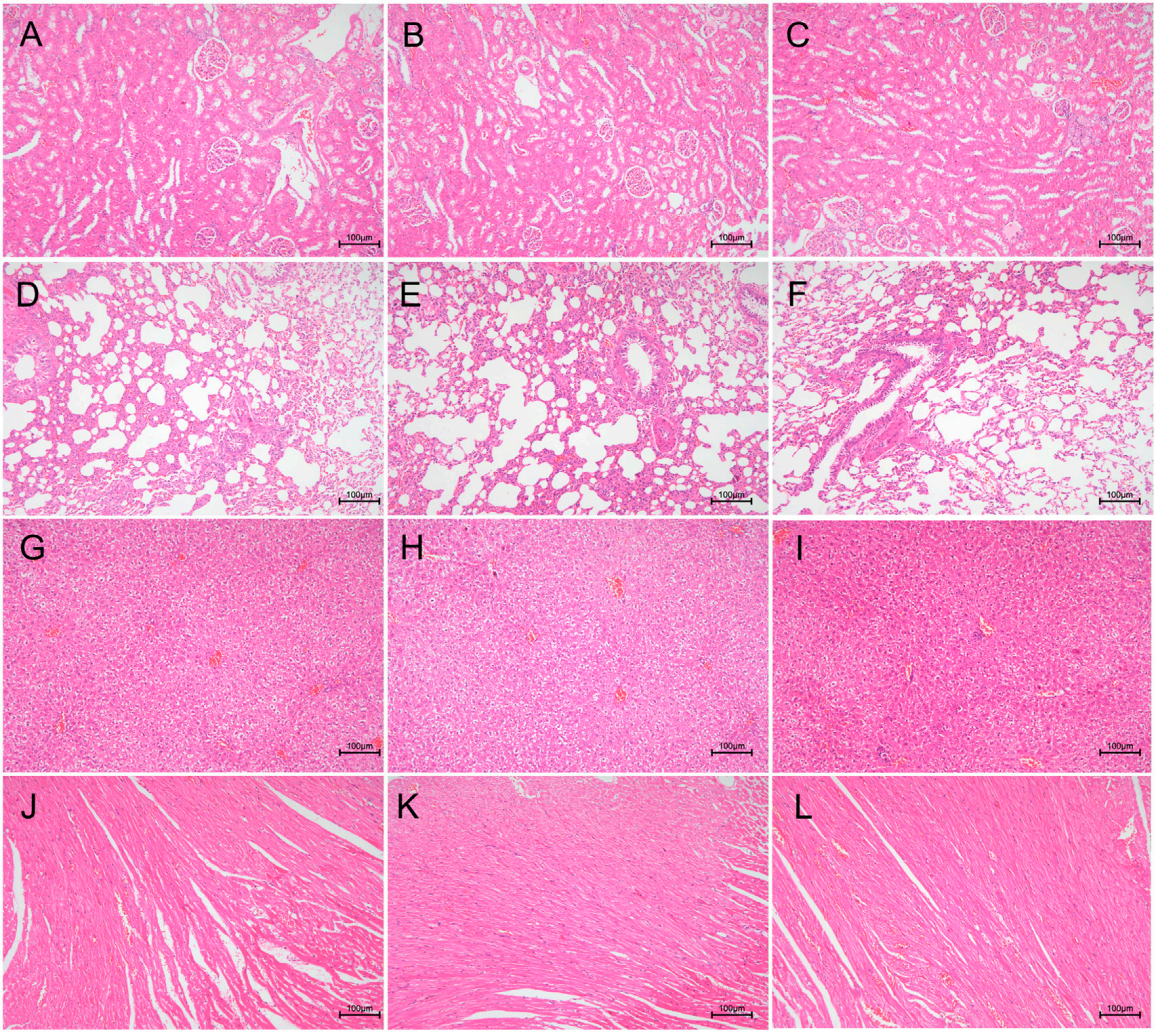
Biocompatibility analysis of (MMT/HA)_10_ and (MMT/HA-RFP)_10_ multilamellar films. H&E staining of rat kidney, lung, liver, and heart. **(A–C)** Kidney in the unmodified, (MMT/HA)_10_, and (MMT/HA-RFP)_10_ groups, respectively. **(D–F)** Lung in the unmodified, (MMT/HA)_10_, and (MMT/HA-RFP)_10_ groups, respectively. **(G–I)** Liver in the unmodified, (MMT/HA)_10_, and (MMT/HA-RFP)_10_ groups, respectively. **(J–L)** Heart in the unmodified, (MMT/HA)_10_, and (MMT/HA-RFP)_10_ groups, respectively.

To evaluate the biocompatibility of the (MMT/HA-RFP)_10_ multilamellar membrane structure, H&E-stained vital organ sections were subjected to histological examination. Heart, liver, lung, and kidney sections from the (MMT/HA)_10_ and (MMT/HA-RFP)_10_ groups exhibited normal morphology (Figure 6). Therefore, the (MMT/HA-RFP)_10_ multilamellar membrane structure demonstrated excellent biocompatibility.

### 3.7 Measurement of body temperature and inflammatory index *in vivo*

**FIGURE 7:**
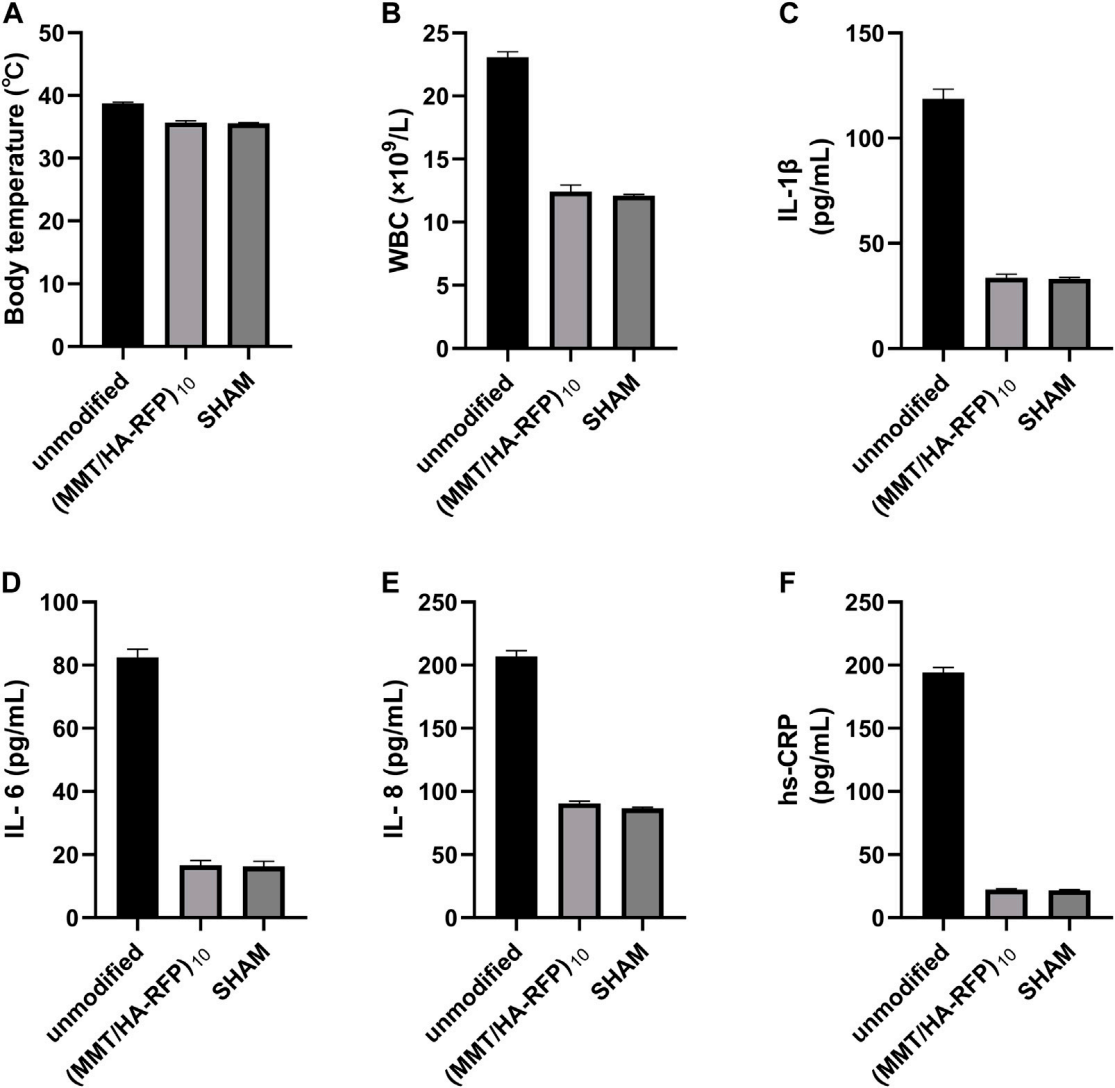
**(A)** Body temperature, **(B)** WBC count, **(C)** IL-1β level, **(D)** IL-6 level, **(E)** IL-8 level, and **(F)** hs-CRP level in rats.

A rat infection model was used to explore the *in vivo* antibacterial properties of (MMT/HA-RFP)_10_ multilamellar films. Body temperature and WBC count, as well as levels of hs-CRP, IL-1β, IL-6, and IL-8, were examined to characterize infection control. At 4 weeks after surgery, body temperature in the sham group was normal (35.57°C ± 0.09°C), whereas body temperature in the unmodified group had significantly increased to 38.73°C ± 0.17°C. However, body temperature in the (MMT/HA-RFP)_10_ group was 35.67°C ± 0.25°C (Figure 7A), presumably because the (MMT/HA-RFP)_10_ multilamellar film released antibiotics that killed bacteria; no rats in that group exhibited postoperative infection. Moreover, WBC count in the unmodified group was elevated at 4 weeks after surgery but within normal limits in the (MMT/HA-RFP)_10_ group (Figure 7B). Similar trends were observed in the levels of IL-1β (Figure 7C), IL-6 (Figure 7D), IL-8 (Figure 7E) and hs-CRP (Figure 7F).

### 3.8 Colonies isolated from K-wires and soft tissue

**FIGURE 8:**
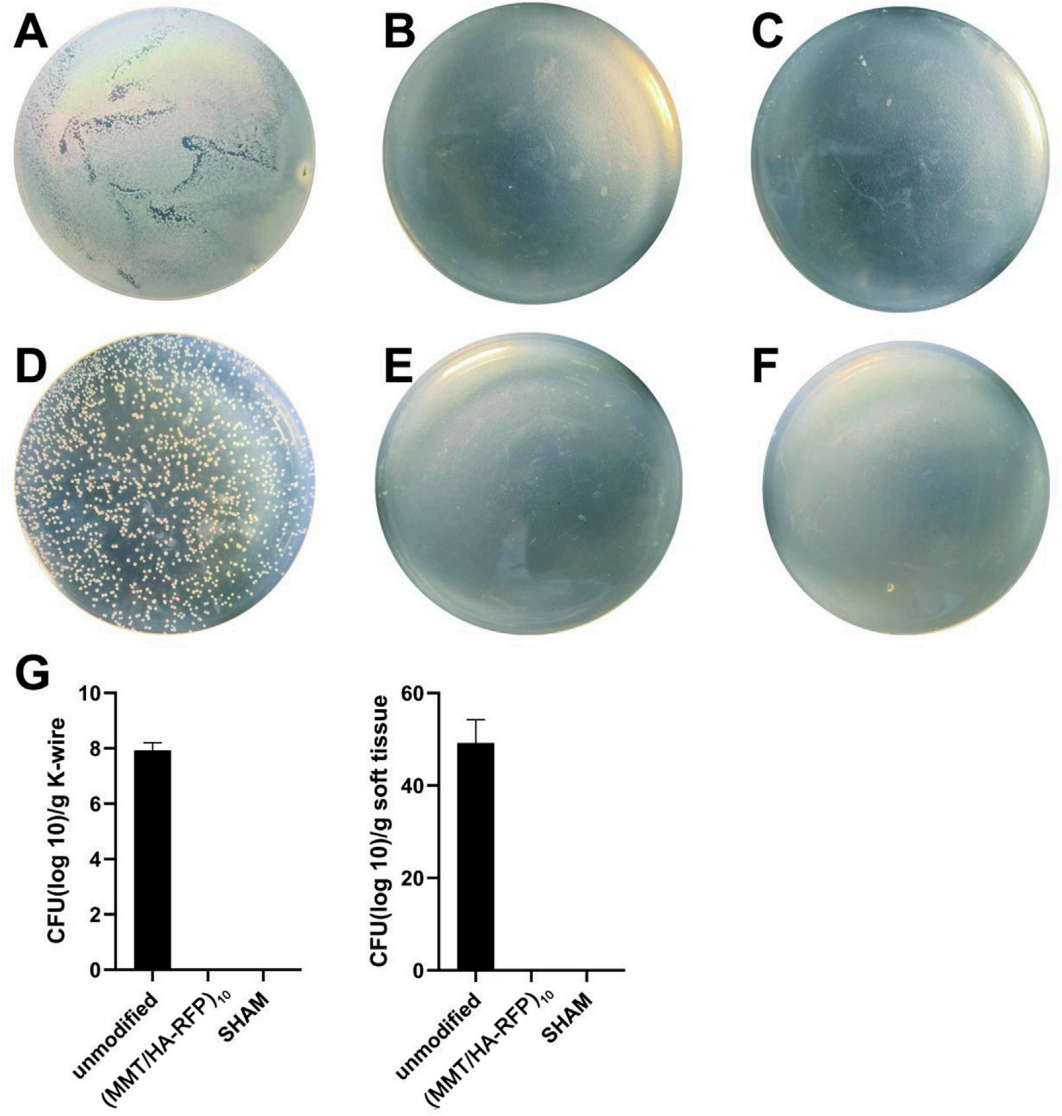
Colonies from K-wires: **(A–C)** unmodified, (MMT/HA-RFP)_10_, and sham groups, respectively. Colonies from soft tissue: **(D–F)** unmodified, (MMT/HA-RFP)_10_, and sham groups, respectively. **(G)** Number of colonies in each group.

Bacteriological examination revealed large amounts of bacteria on K-wires and in soft tissue in the unmodified group (Figures 8A,D), indicating that bacteria spread *via* circulation and multiplied throughout the body. However, there were no bacteria in the (MMT/HA-RFP)_10_ group (Figures 8B,E). As expected, there were also no bacteria in the sham group (Figures 8C,F). Compared with the unmodified group, the surfaces of the K-wires and soft tissue in the (MMT/HA-RFP)_10_ group showed 7.93 ± 0.23 and 49.20 ± 4.12 log reductions of *S. aureus*, respectively (Figure 8G).

### 3.9 X-ray and CT examination

**FIGURE 9:**
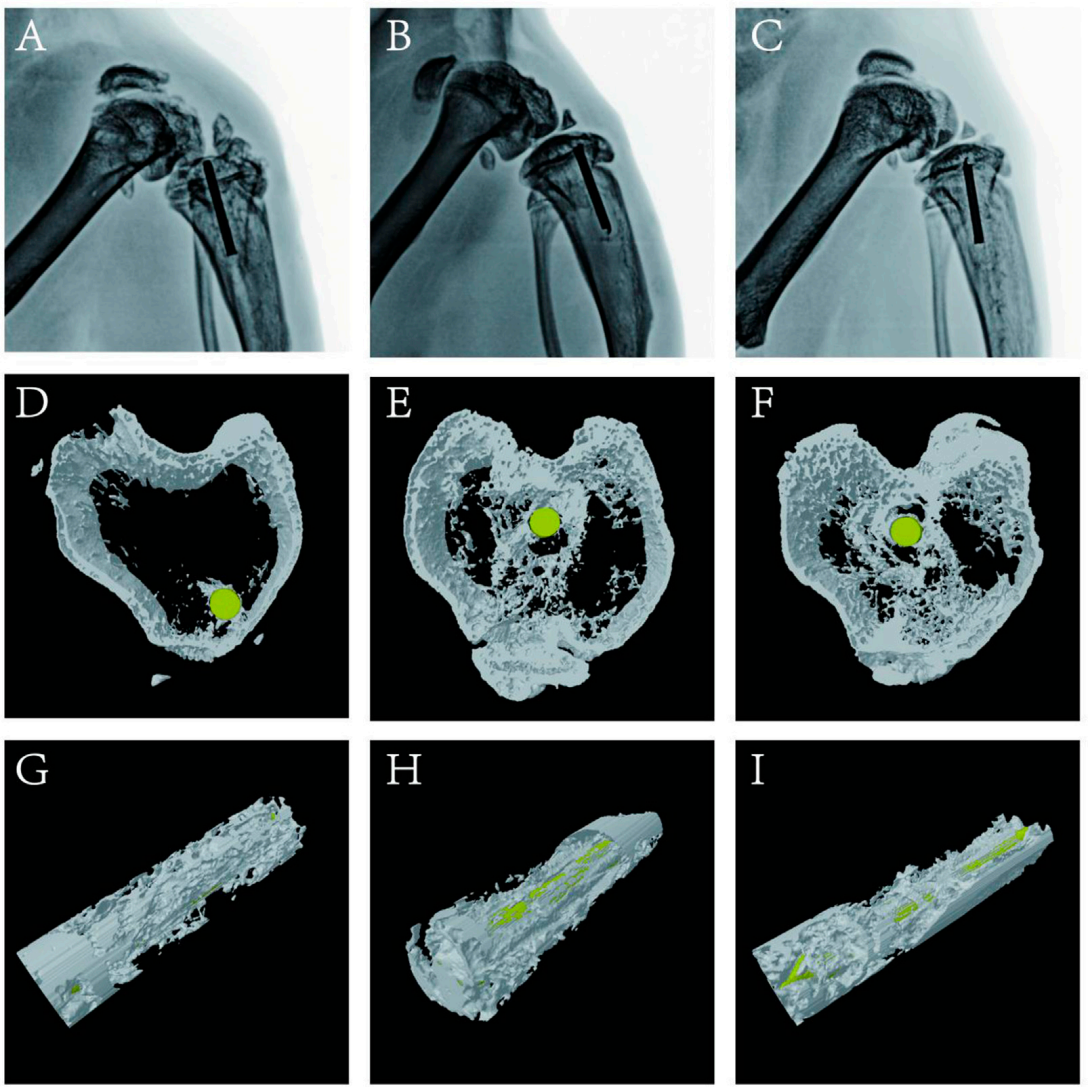
X-ray examination: **(A–C)** unmodified, (MMT/HA-RFP)_10_, and sham groups, respectively. Trabecular distribution: **(D–F)** unmodified, (MMT/HA-RFP)_10_, and sham groups, respectively. New bone formation surrounding implants: **(G–I)** unmodified, (MMT/HA-RFP)_10_, and sham groups, respectively.

**FIGURE 10:**
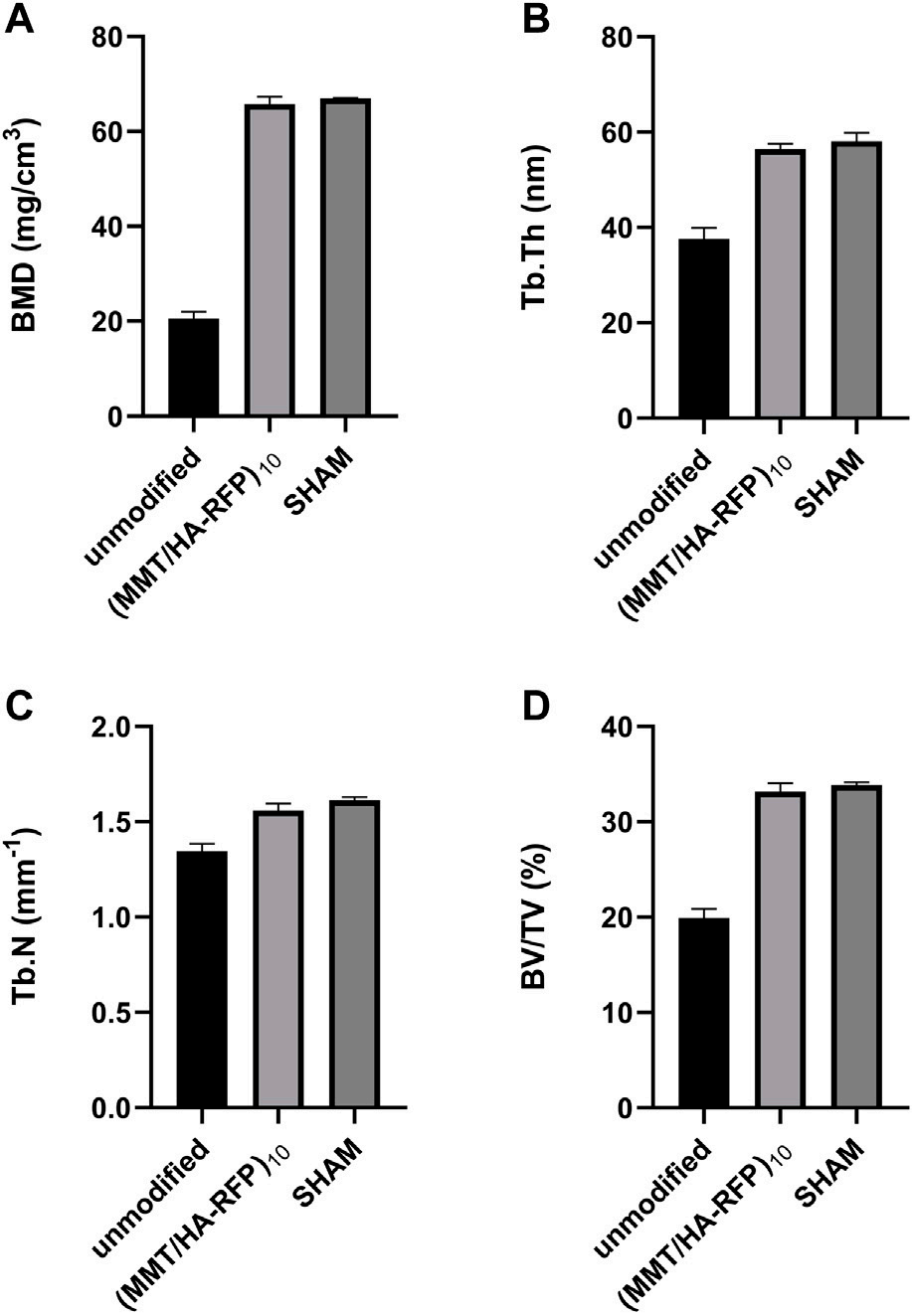
Analysis of **(A)** BMD, **(B)** Tb.Th, **(C)** Tb.N, and **(D)** BV/TV in each group.

Bone infection is commonly assessed by X-ray examination in clinical practice (Mistry et al., 2016). Bone in the sham group exhibited excellent structural integrity (Figure 9C), confirming that the surgeries had been performed successfully, animal management was reliable, and no infections had occurred. The unmodified group exhibited bone destruction on the tibial plateau, and the femoral condyle was infected (Figure 9A). These phenomena may have occurred because bacteria multiplied throughout the body and severely damaged the bone structure. Notably, no bone destruction was observed in the (MMT/HA-RFP)_10_ group, despite injection of *S. aureus* into the bone (Figure 9B). To comprehensively assess bone tissue microstructure, three-dimensional histograms were constructed using the software provided with the CT scanner. K-wires appeared yellow on cross-sections of rat tibia. Trabecular bone constitutes the internal extension of cortical bone in the bone marrow cavity. There is an irregular network structure inside, which supports hematopoietic function; this microstructure is extremely difficult to segment within the bone (Kesavan et al., 2017). As shown in Figure 9D, the unmodified group exhibited the widespread rupture and disordered arrangement of trabecular bone. However, the (MMT/HA-RFP)_10_ and sham groups exhibited considerably less trabecular bone destruction (Figures 9E,F). Moreover, minimal new bone formation was visible in the unmodified group (Figure 9G). In contrast, a considerable amount of new bone was detected around implants in the (MMT/HA-RFP)_10_ group; the new bone was in close contact with the implant surface (Figure 9H). Importantly, the findings were similar between the (MMT/HA-RFP)_10_ and sham groups (Figure 9I). Furthermore, micro-CT was performed to quantify BMD, Tb.N, BV/TV, and Tb.Th (Figure 10). In the sham group, the tibia lesions demonstrated normal values of BMD, Tb.N, BV/TV, and Tb.Th. In the unmodified group, the presence of bacteria led to significant decreases in the tibial total BMD, Tb.N, BV/TV, and Tb.Th. Bacterial proliferation affects bone cell growth (Min et al., 2016). Surprisingly, all characteristics in the (MMT/HA-RFP)_10_ group resembled characteristics in the sham group.

### 3.10 Bending tests of tibia specimens

**FIGURE 11:**
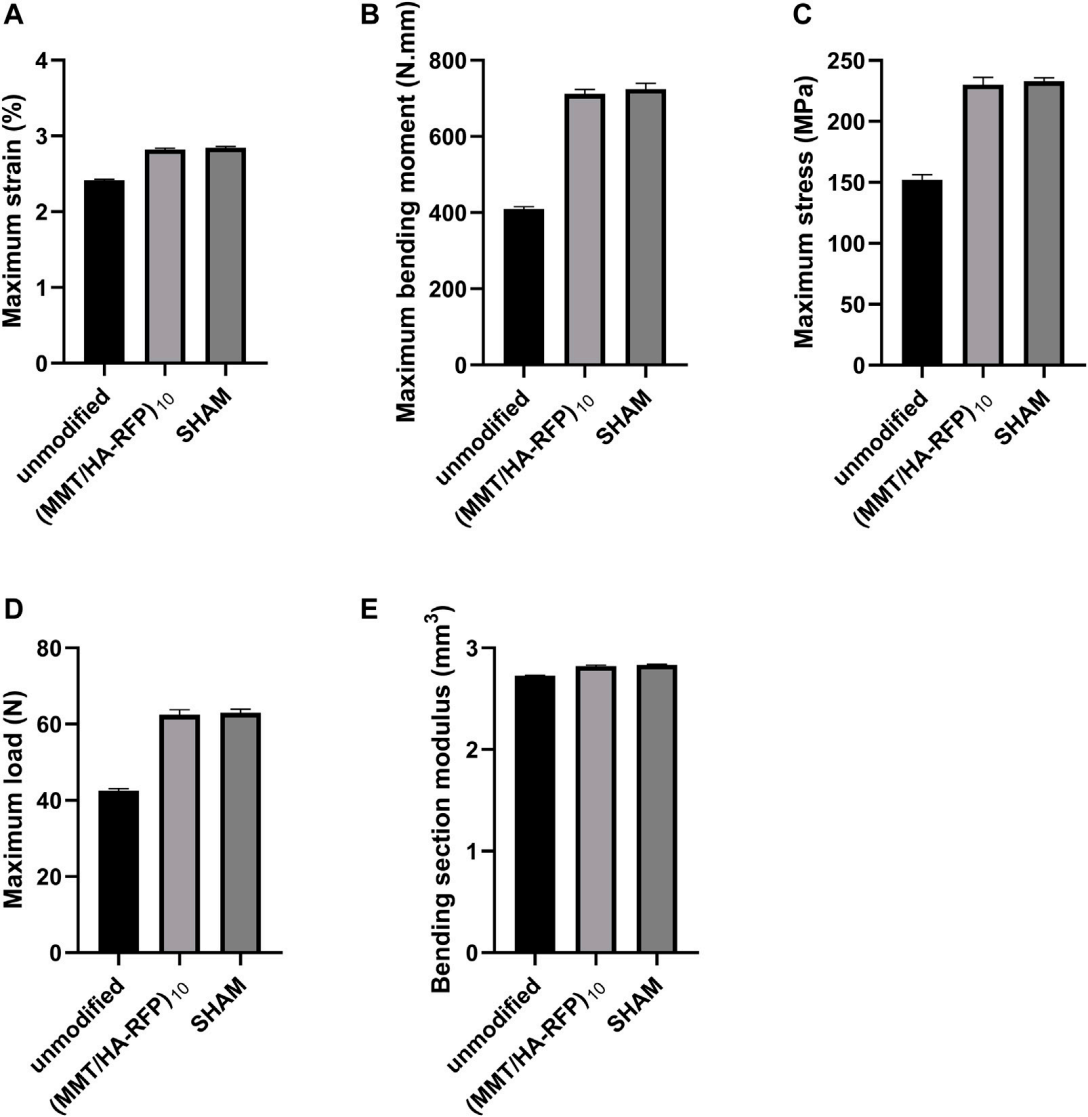
Bending tests of tibia specimens: **(A)** maximum strain, **(B)** maximum bending moment, **(C)** maximum stress, **(D)** maximum load, **(E)** bending section modulus.

The three-point bending test was used to examine the integration strength of bone samples in each group through assessments of maximum load, maximum strain, maximum bending moment, maximum stress, and bending section modulus. As shown in Figure 11, tibia lesions in the sham group had normal values for maximum load, maximum strain, maximum bending moment, maximum stress, and bending section modulus. However, all values were significantly lower in the unmodified group. Bacteria can reportedly disrupt homeostatic balance in bone and destroy the integrity of bone microstructure (Raphel et al., 2016). Importantly, all characteristics in the (MMT/HA-RFP)_10_ group resembled characteristics in the sham group, confirming that the (MMT/HA-RFP)_10_ multilamellar membrane structure has good antibacterial properties.

### 3.11 Histological analysis

**FIGURE 12:**
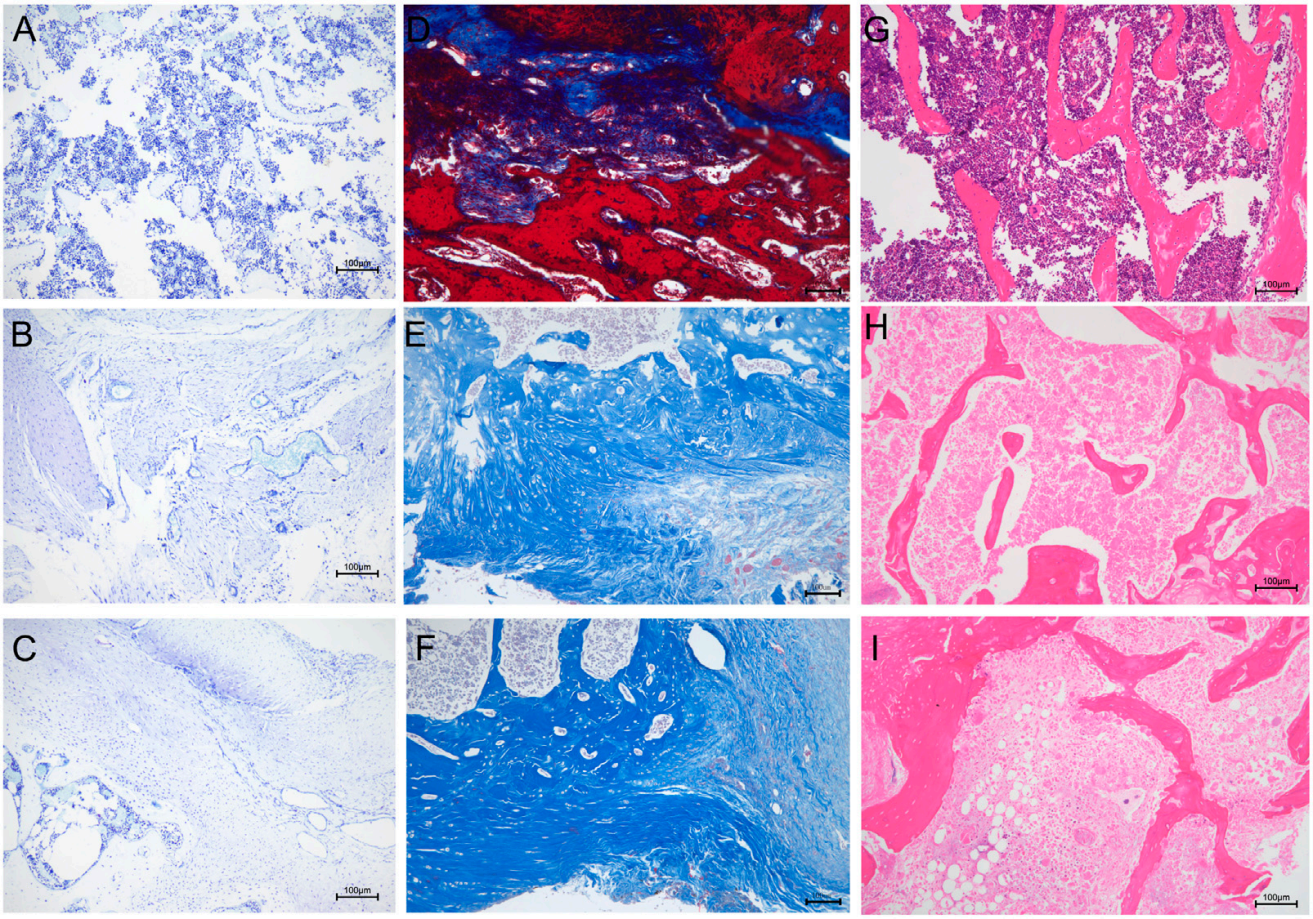
Histological analysis. Toluidine blue staining: **(A–C)** unmodified, (MMT/HA-RFP)_10_, and sham groups, respectively. Masson’s trichrome staining: **(D–F)** unmodified, (MMT/HA-RFP)_10_, and sham groups, respectively. H&E staining: **(G–I)** unmodified, (MMT/HA-RFP)_10_, and sham groups, respectively.

We performed further histological analyses by staining bone sections with H&E, Masson’s trichrome, and toluidine blue. Toluidine blue staining showed numerous mast cells in the bone tissue of the unmodified group (Figure 12A). Mast cells can release large amounts of inflammatory mediators, such as tumor necrosis factor-α, IL-1, and IL-8 (Delgado et al., 2020). However, inflammatory mast cell infiltration was significantly reduced in the (MMT/HA-RFP)_10_ group (Figure 12B). In the sham group, mast cells were not observed, and the bone tissue structure was normal (Figure 12C). Furthermore, the sections were subjected to Masson’s trichrome staining to visualize collagen fibers and assess fibrotic lesions in bone tissue (Griffin et al., 2019). In the unmodified group, most areas were stained red because of infection-induced fibrosis in the bone marrow cavity (Figure 12D). Notably, bone tissue was completely free of fibrotic lesions in the (MMT/HA-RFP)_10_ and sham groups (Figures 12E, 2F). H&E staining revealed that large quantities of inflammatory cells had penetrated the bone trabecula in the unmodified group, which confirmed the presence of bone infection (Figure 12G). However, no inflammatory responses (e.g., swelling or inflammatory cell infiltration) were found in the (MMT/HA-RFP)_10_ and sham groups (Figures 12H,I).

## 4 Conclusion

In this study, a (MMT/HA-RFP)_10_ multilamellar film structure was successfully prepared from MMT, HA, and RFP; its thickness increased linearly during layer-by-layer assembly. The (MMT/HA-RFP)_10_ multilamellar film structure gradually deteriorated and exhibited concentration-dependent degradation during incubation with solutions containing HAS and *S. aureus*. The (MMT/HA-RFP)_10_ multilamellar film structure showed good antibacterial properties, as determined by analyses of biofilm formation and shaking flask culture. The hemolysis rate highlighted the excellent biocompatibility of this material. (MMT/HA-RFP)_10_ multilamellar film-coated K-wires showed excellent antibacterial properties and excellent biocompatibility *in vivo*. Further studies are needed to fully characterize the clinical potential of this material.

## Data Availability

The original contributions presented in the study are included in the article/Supplementary Material; further inquiries can be directed to the corresponding author.
